# SPIO labeling of endothelial cells using ultrasound and targeted microbubbles at diagnostic pressures

**DOI:** 10.1371/journal.pone.0204354

**Published:** 2018-09-20

**Authors:** Ilya Skachkov, Ying Luan, Sandra T. van Tiel, Antonius F. W. van der Steen, Nico de Jong, Monique R. Bernsen, Klazina Kooiman

**Affiliations:** 1 Department of Biomedical Engineering, Thoraxcenter, Erasmus MC, Rotterdam, the Netherlands; 2 Department of Radiology & Nucleair Medicine, Erasmus MC, Rotterdam, the Netherlands; 3 Laboratory of Acoustical Wavefield Imaging, Faculty of Applied Sciences, Delft University of Technology, Delft, the Netherlands; Monash University, AUSTRALIA

## Abstract

*In vivo* cell tracking of therapeutic, tumor, and endothelial cells is an emerging field and a promising technique for imaging cardiovascular disease and cancer development. Site-specific labeling of endothelial cells with the MRI contrast agent superparamagnetic iron oxide (SPIO) in the absence of toxic agents is challenging. Therefore, the aim of this *in vitro* study was to find optimal parameters for efficient and safe SPIO-labeling of endothelial cells using ultrasound-activated CD31-targeted microbubbles for future MRI tracking. Ultrasound at a frequency of 1 MHz (10,000 cycles, repetition rate of 20 Hz) was used for varying applied peak negative pressures (10–160 kPa, i.e. low mechanical index (MI) of 0.01–0.16), treatment durations (0–30 s), time of SPIO addition (-5 min– 15 min with respect to the start of the ultrasound), and incubation time after SPIO addition (5 min– 3 h). Iron specific Prussian Blue staining in combination with calcein-AM based cell viability assays were applied to define the most efficient and safe conditions for SPIO-labeling. Optimal SPIO labeling was observed when the ultrasound parameters were 40 kPa peak negative pressure (MI 0.04), applied for 30 s just before SPIO addition (0 min). Compared to the control, this resulted in an approximate 12 times increase of SPIO uptake in endothelial cells *in vitro* with 85% cell viability. Therefore, ultrasound-activated targeted ultrasound contrast agents show great potential for effective and safe labeling of endothelial cells with SPIO.

## Introduction

*In vivo* cell tracking is a very promising technique to visualize cells of interest inside the body. It allows tracking of motile therapeutic cells like immune cells, stem cells, and endothelial progenitor cells to sites of inflammation, cancer, or ischemia [1–5]. Additionally, this technique can be used to track tumor cells [6], tumor vasculature [7, 8], or endothelial cells in tissue engineered valves [9] and vascular grafts [10].

After labeling the cells of interest with an imaging probe, they can be tracked by an imaging modality. Magnetic resonance imaging (MRI) is interesting for cell tracking because it is precise, harmless, and thus well suited for longitudinal studies. Moreover, single cell tracking is possible by MRI. However, *in vivo* cell labeling with an MRI contrast agent is challenging [6, 11–16]. For cell labeling, the T2 and T2*-shortening MRI contrast agent superparamagnetic iron oxide nanoparticles (SPIO) of 80–180 nm in size [17] are often used [18, 19]. They are relatively safe compounds [19–22], but most of *in vitro* cell labeling techniques for SPIO are not applicable *in vivo*, because of the high toxicity and broad systemic effects of transfection agents [23]. Therefore, there has been growing interest in safe, site-specific cell labeling techniques. One potential method involves using ultrasound contrast agent, which are comprised of microbubbles. The microbubbles have a low diffusible gas core (for example C_4_F_10_), vary in diameter from 1–10 μm, and are encapsulated by a coating material (for example phospholipids). When ultrasound is applied, the microbubbles oscillate due to sequential compression and expansion caused by pressure variations in the surrounding medium. The oscillation of microbubbles has been shown to deliver therapeutic materials into cells and interstitial tissue [24–27]. Up till now there is no consensus on the mechanism of the enhanced uptake. One of the uptake routes is a phenomenon called sonoporation, when reversible or non-reversible cell membrane pores are generated upon microbubble oscillations or violent collapse. Other uptake routes include enhanced endocytosis and opening of cell-cell contacts [25, 26, 28]. It has been reported that the efficacy of cellular uptake of therapeutic agents can be improved up to 7.7-fold *in vitro* [29] and up to fivefold *in vivo* [30] by using targeted microbubbles (tMB) instead of non-targeted microbubbles (non-tMB). The tMB have a ligand added in their coating by which the tMB can adhere to disease-specific cell membrane biomarkers [31, 32].

It was previously shown that 45–60 nm SPIO (Resovist) could be delivered *in vivo* into the swine brain using SonoVue lipid-coated non-tMB and ultrasound (28-kHz ultrasound with 100-ms burst length and repetition rate of 1 Hz at 0.6–1 MPa (mechanical index (MI) 4.8–6.0) applied for 5 min; MRI performed 3 h after treatment) [33]). Brain tumor delivery of SPIO (mean diameter 6–10 nm [34] or 35.7 ± 9.2 nm [35]) loaded in the lipid-coating of in-house produced non-tMB was shown *in vivo* in rats using ultrasound (0.4 MHz with 1,000 cycles and repetition rate of 1 Hz at 325 kPa (MI 0.5) applied for 90 s; MRI performed 40 min after treatment [34] or 1 MHz with 5,000 cycles and repetition rate of 1 Hz at 300 kPa (MI 0.3) applied for 4 min; MRI performed 1 and 3 h after treatment [35]). Delivery of 120–180 nm SPIO (Feridex) was also shown in the aortic arch by SonoVue and ultrasound treatment (8.5 MHz ultrasound at an MI of 1.2 applied for 20 min; MRI performed 1 h after treatment) [36]. These studies demonstrate the possibility of SPIO-loaded MB or co-administrated SPIO with MB for labeling extravascular tissues and subsequent MRI imaging of the SPIO, but do not cover cell labeling. Successful SPIO (Revovist) mesenchymal stem cell labeling using SonoVue and ultrasound (1 MHz, 50% duty cycle, 1.0 W/cm^2^ acoustic power applied for 60 s) has been reported *in vitro* [37]. SPIO (12 nm mean diameter) loaded in the polymer coating of in-house produced non-tMB were used to successfully label tumor cells *in vitro* using ultrasound (1 MHz, 20 cycles per burst, repetition rate of 10 kHz, 0.1–0.75 W/cm^2^ acoustic power applied for 40 s) [38]. However, MB are blood pool agents. Endothelial cells, which form the inner lining of vessels, are therefore the main target of intravascular administered MB [39, 40]. Exceptions are tumors that invade into the vasculature, as reported for hepatocellular cancer (i.e. a primary liver tumor) [41] and colorectal cancer [42]. On the other hand, tMB were shown to target ovarian cancer cells preclinically by an alternative administration route, namely intraperitoneal injection [43]. Additionally, tMB are preferable instead of non-tMB since they can be specifically targeted to the cells of interest and upon binding are close to the endothelium, which is a perquisite for the MB-mediated drug delivery effectiveness [26]. The *in vivo* study by Gao et al. demonstrated arterial wall uptake of SPIO particles using non-tMB and ultrasound [36], but only under one acoustic setting (8.5 MHz ultrasound at 1.2 MI, i.e. 3.5 MPa acoustic pressure), which induced considerable arterial wall damage. To the best of our knowledge, no in-depth studies have been performed to characterize the parameters (e.g., the acoustic settings, the SPIO addition time and incubation time) that strongly influence the efficacy and safety of SPIO-labeling of endothelial cells using tMB at low MI (<0.2).

The aim of our *in vitro* study was to find optimal parameters for non-invasive, tMB-mediated, SPIO-labeling of endothelial cells for the future application of MRI tracking of tumor vasculature and tissue engineered vasculature structures. We used lipid-coated tMB targeted against CD31 (i.e. platelet/endothelial cell adhesion molecule-1 (PECAM1)), a biomarker constitutively expressed on endothelial cell membranes [44], as proof of concept. Iron specific Prussian Blue staining in combination with calcein-AM based cell viability assays were applied to define the most efficient and safe conditions for SPIO-labeling of endothelial cells *in vitro*. We investigated a fixed ultrasound driving frequency of 1 MHz and a series of low diagnostic acoustical pressures (<200 kPa; MI<0.2) and treatment duration times (0–30 s). In our study we used 1 MHz as the ultrasound frequency because it is commonly used for microbubble-mediated drug delivery studies and is close to the resonance frequency of microbubbles [26]. Although the exact link between the type of microbubble behavior and drug uptake is not yet known [26], it was reported that endocytosis was stimulated at longer (2,000–10,000 cycles) acoustic cycles [45–47]. SPIO are typically 80–150 nm [17] nanoparticles which may require uptake by endocytosis, as this has been shown to be the main uptake mechanism for therapeutics larger than ~17 nm in radius [46]. This is the reason why we chose to study 10,000 acoustic cycles.

## Materials and methods

### Endothelial cells

Human umbilical vein endothelial cells (HUVECs) (Lonza, Verviers, Belgium) were cultured in EGM-2 (Lonza) medium in T75 flasks (BD, Breda, the Netherlands) in a humidified incubator at 37°C with 5% CO_2_. Cells were detached with 0.25% Trypsin in EDTA (Lonza) and replated on one side of the acoustically transparent OptiCell^™^ (NUNC, Wiesbaden, Germany) chambers. HUVECs were cultured as described before [48], for two days until 70% confluence to resemble neovasculature endothelial cells.

### Targeted microbubbles

Biotinylated lipid-coated microbubbles (mean diameter 2.5 μm) consisting of a coating of DSPC (59.4 mol %; P 6517; Sigma-Aldrich, Zwijndrecht, the Netherlands), PEG-40 stearate (35.7 mol %; P 3440; Sigma-Aldrich), DSPE-PEG(2000) (4.1 mol %; 880125 P; Avanti Polar Lipids, Alabaster, AL, USA), and DSPE- PEG(2000)-biotin (0.8 mol %; 880129 C; Avanti Polar Lipids) with a perfluorobutane (C_4_F_10_) gas core (F2 Chemicals, Preson, UK) were made by sonication as previously described [49, 50]. Biotinylated anti-human CD31-antibody (BAM3567; R&D Systems, Europe, Abingdon, United Kingdom) was conjugated to the microbubbles via avidin-biotin bridging as previously described [50, 51]. Specificity of binding of these CD31-targted microbubbles was previously reported by us [48].

### Cell treatment

**Fig 1:**
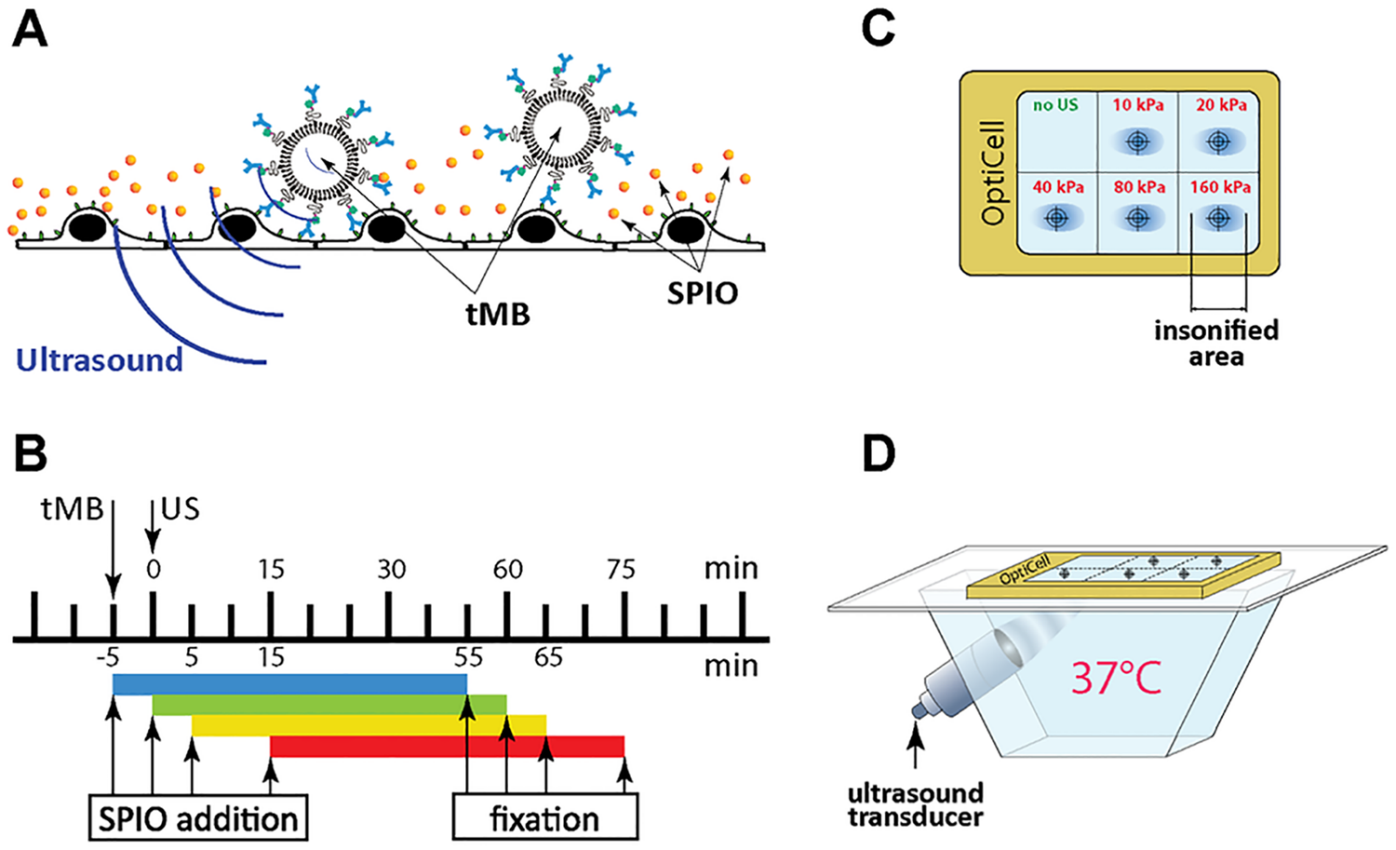
Experimental setup. (A) Schematic representation of the tMB adhering to HUVECs during treatment. (B) Timing diagram of the experiment. The time of insonification (0 min) was used as the reference time. Targeted microbubbles (tMB) were added 5 min before the ultrasound was applied; SPIO was added 5 min before (i.e. -5 min), just before (i.e. 0 min), 5 min after, and 15 min after insonification. Cells were fixated 60 min after SPIO addition. (C) Scheme of insonification of subsections of the OptiCell^™^ chamber (to scale). The acoustic pressure is given in PNP. (D) The treatment setup.

The concentration of tMB was evaluated by Coulter Counter (Multisizer 3, Beckman Coulter, Mijdrecht, the Netherlands) measurements (n = 3) using a 20-μm aperture tube allowing quantification of particle diameters between 0.4 and 12 μm using a linear spacing between the 256 channels. Ten million tMB were added to an OptiCell^™^ chamber with cells plated on the bottom (cell to bubble ratio of 1:3), which was turned upside down to let microbubbles adhere to the cells by flotation. After 5 min incubation at 37°C, the chamber was reverted for the experiment so the bound tMB were on top of the endothelial cells as shown in Fig 1A. SPIO nanoparticles (Endorem^™^, Gerber S.A., Paris, France) were added at four time-points: 5 min before, immediately before (0 min), 5 min after, and 15 min after insonification as illustrated in Fig 1B, at a final concentration of 22.4 μg Fe/ml. Each OptiCell^™^ chamber was divided into six acoustically non-overlapping areas (25 × 30 mm each; see Fig 1C), which covered the beam area (6.5 mm for -6dB beam width) at the focus of the 1.0 MHz transducer (V303; Panametrics-NDTTM, Olympus NDT, Waltham, MA, USA), as verified in advance with a calibrated 0.2 mm PVDF needle hydrophone (Precision Acoustics Ltd, Dorchester, UK). The OptiCell chamber was placed into a 37 °C water bath and connected to a 2D micropositioner (Fig 1D). The 1 MHz focused transducer was configured at a 45° angle below the sample and the acoustic focus was aligned with the center of each subsection.

During the experiment, the position of the OptiCell chamber was manipulated so that the center of each subsection was insonified in sequence at a predefined pressure (10 to 160 kPa peak negative pressure (PNP), Fig 1C). A prolonged burst of 10,000 cycles with a repetition rate of 20 Hz was applied generated by an arbitrary waveform generator (33220A, Agilent, Palo Alto, CA, USA) and amplified using a broadband amplifier (ENI A-500, Electronics & Innovation, Rochester, NY, USA). The first subsection, without the application of ultrasound, was used as the control. The effect of the different total insonification time was determined (1 s, 10 s, and 30 s) at 40, 80, and 160 kPa PNP when SPIO were added 5 min prior to insonfication. To investigate the effect of the incubation time with SPIO, the OptiCells were incubated at 37°C for 5 min, 1 h, and 3 h after insonification when SPIO were added 5 min prior to insonification. The effect of SPIO addition time (-5, 0, 5, and 15 min with respect to the start of insonification) was determined at 10, 20, 40, 80, and 160 kPa PNP. To check the effect of insonification of HUVECs on SPIO uptake in the absence of tMBs, the OptiCells were insonified at 40, 80, and 160 kPa PNP for 30 s, while SPIO was added 5 min prior to insonification (n = 2). All other experiments were repeated three times. From these three datasets the average and standard deviation are plotted.

### SPIO labeling

After the treatment described above (see also Fig 1B), cells were rinsed three times with phosphate-buffer saline (PBS; Invitrogen, Groningen, the Netherlands) to remove non-internalized SPIO. Then, cells were fixated with 4% formaldehyde (Sigma-Aldrich, Zwijndrecht, the Netherlands) for 10 min. After fixation, the cells were washed three times with PBS and then incubated with Prussian Blue solution for 30 min (aqueous solution of 10% hydrochloric acid (Sigma-Aldrich) and 5% potassium ferrocyanide (Sigma-Aldrich)) to assess the SPIO-labeling [52]. Next, the cells were washed three times with PBS and the nuclei were stained with 0.1% nuclear fast red solution (Sigma-Aldrich). Thereafter the OptiCells were dried for 48 h and microscopically examined using a microscope (Olympus, Zoeterwoude, the Netherlands) equipped with 20× Plan (NA 0.4) objective (Olympus) and a color camera (Axiocam MRc, Carl Zeiss, Germany). SPIO uptake was assessed by manually counting Prussian Blue positive cells among ~500 cells (acquired in 5 fields of view) located within a circle of 6 mm diameter around the center point of each insonified area. A cell was counted as SPIO positive when it contained one or more Prussian Blue stained iron particles.

### Cell viability assay

For each SPIO uptake measurement, cell viability was determined in triplicate by calcein-AM and propidium iodide (PI) assays in parallel. Cells were treated with SPIO, tMB and ultrasound as described before (see also Fig 1). Within 3 to 4 min after the US treatment of all subsections of the Opticells, HUVECs were incubated at 37 °C, 5% CO_2_. Thirty min before assessing the cell viability, calcein-AM was added to the OptiCell chamber (C3100MP; Invitrogen; 0.25 μM final concentration from a 1 mM stock prepared in DMSO (Sigma-Aldrich)) and incubated for 30 min under the same conditions. Thereafter PI (P4864, Sigma-Aldrich, 25 μg/ml final concentration) and Hoechst 33342 (Invitrogen; 5 μg/ml final concentration) were added to the Opticell chamber. Microscopic examination was performed directly after the PI and Hoechst addition with a fluorescent microscope (Olympus) equipped with the same setup as applied for SPIO labeling measurements, only that a 5× LMPlanFl (NA 0.13) objective (Olympus) was used here. For each condition five different fields of view were acquired (~2900 cells) within the 6 mm circle around the center of the insonified area. Different filter sets (U-MWU2, 330–385/420 nm; U-MWIB2, 460–490/510 nm; U-MWG2, 510–550/570 nm, Olympus) were applied for detecting all cells (stained with Hoechst), viable cells (stained with calcein-AM), and dead cells (stained with PI) respectively. All images were automatically analyzed in ImageJ [53]. The Find Maxima function in ImageJ was used to define the exact number of cells. To find an appropriate tolerance for the Find Maxima function in every image, the number of local maxima was defined for tolerance parameters of 0 to 200 in steps of two. We analyzed the differences in number of maxima between the steps. When the difference became smaller than 20, this point was considered as the correct tolerance and the corresponding number of cells as correct number of cells. This approach was validated by selective manual counting of number of cells (n = 10). The difference between manual and automatic counting was 2.1±0.4%. As shown in S1 Fig, the % of viable cells determined from the calcein-AM staining (live cells) was the same as the cell viability determined from the PI staining (dead cells). The cell viability data was therefore presented as the % of viable cells determined from the calcein-AM staining.

## Results

### Microbubble dynamics

**Fig 2:**
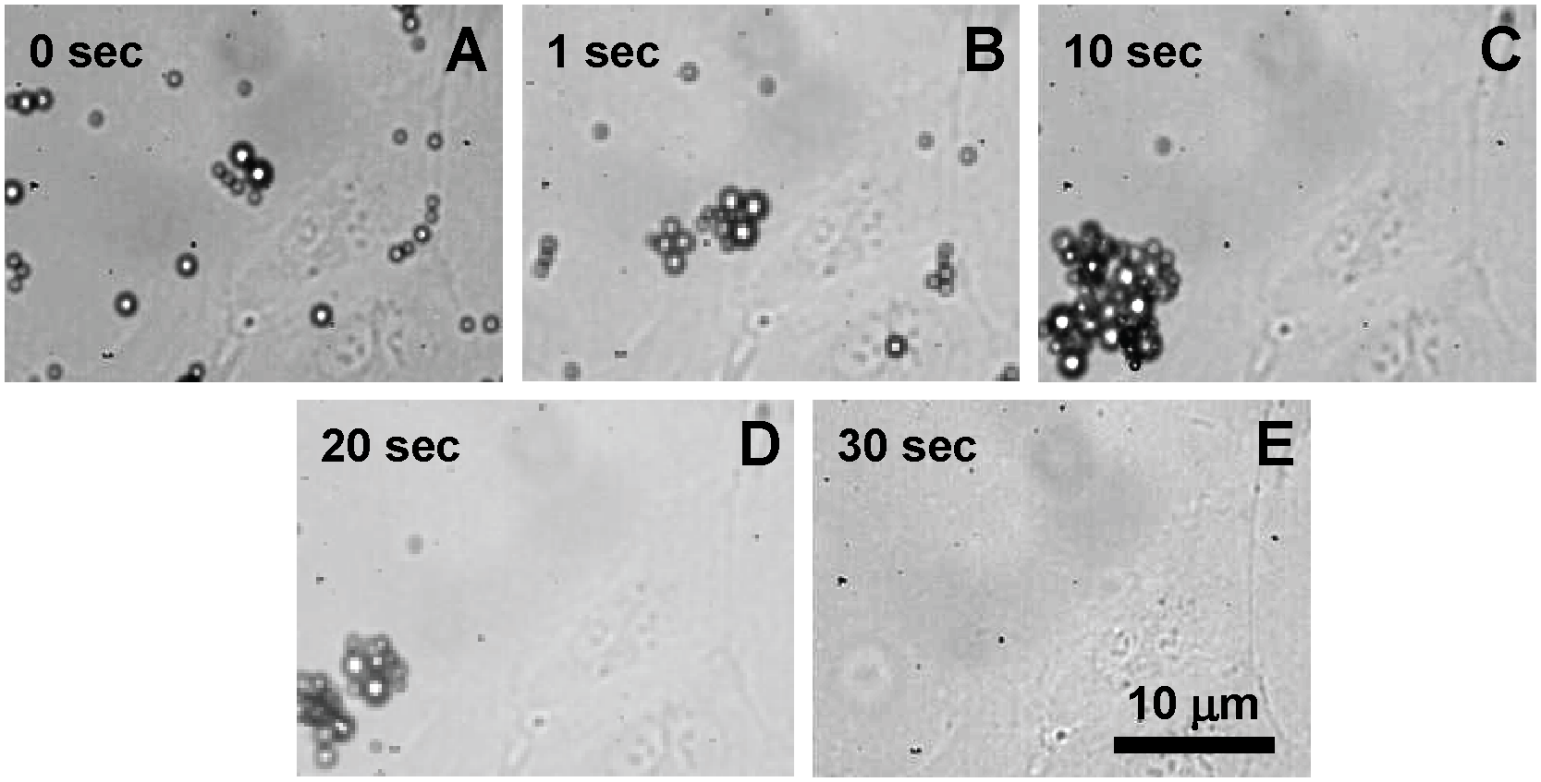
Optical recording of tMB on HUVECs during ultrasound treatment. (A) before treatment. (B-E) tMB displacement, clustering, and destroyment during 30 s insonification (1MHz, 80 kPa PNP, 10,000 cycles, repetition rate of 20 Hz, 30 s insonification treatment, SPIO added at -5 min with respect to the start of insonification).

During all studied ultrasound bursts, we observed displacement and disappearance of tMB. This was most pronounced for the 30 s insonification period, as shown in Fig 2. During insonification, tMB also clustered (Fig 2B–2D). After the 30 s insonification period, no tMB were observed in the field of view (Fig 2E), suggesting they had been destroyed.

### Insonification duration

**Fig 3:**
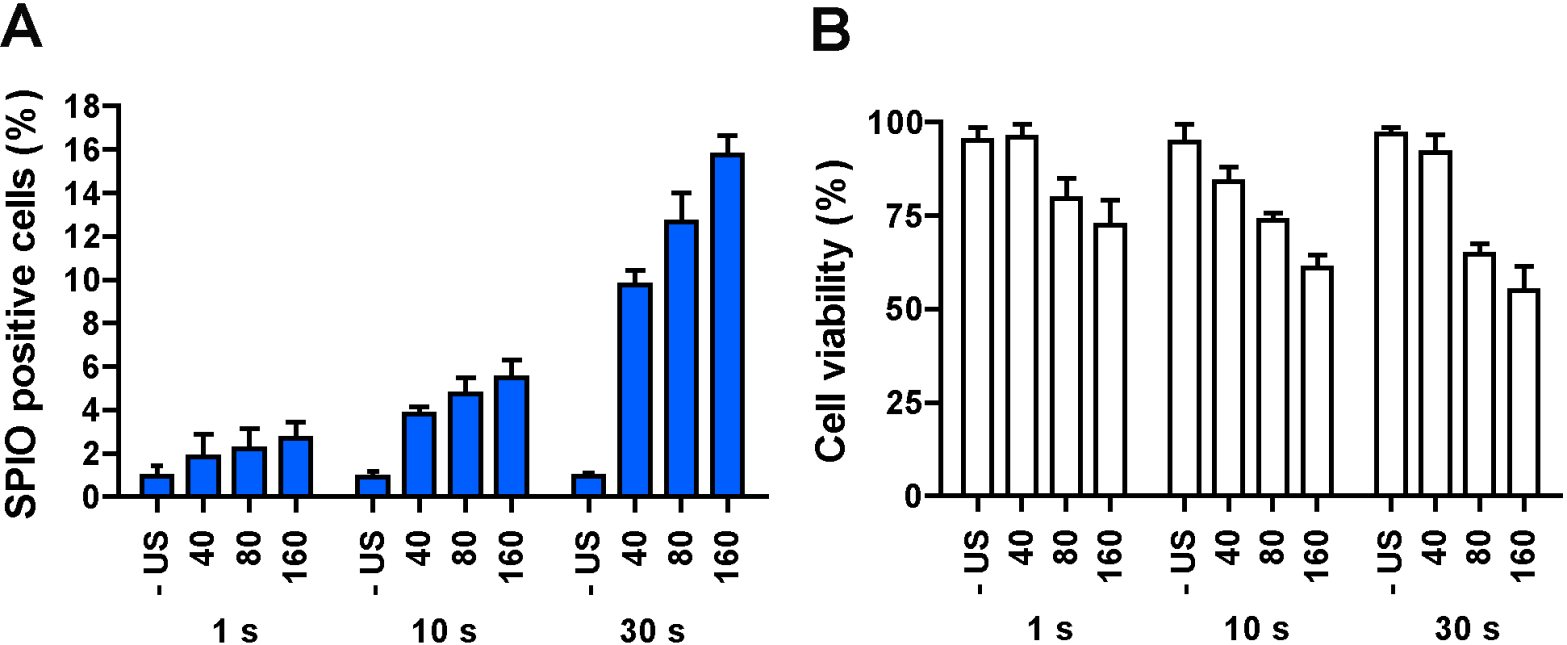
The influence of ultrasound insonification time on intracellular SPIO uptake efficiency. (A) SPIO positive cells. (B) Cell viability. HUVECs were treated with tMB and no ultrasound (- US) or ultrasound at varying PNP (40, 80, or 160 kPa) for 1, 10, or 30 s; SPIO added 5 min before insonification (-5 min); 1 h of incubation after SPIO addition.

In the absence of ultrasound, less than 2% of cells intracellularly incorporated SPIO naturally (Fig 3A). The efficacy of SPIO uptake by HUVECs and the corresponding cell viability as a function of the acoustic PNP and the total insonification duration (1, 10, or 30 s) at low MI (<0.16) are shown in Fig 3. The total ultrasound exposure time was a key factor for SPIO uptake efficacy. This was demonstrated by the amount of SPIO positive cells not exceeding 4% for 1 s and 6% for 10 s of insonification, but with 30 s of insonification the amount of SPIO positive cells increased to more than 10%. Additionally, the PNP also influenced SPIO uptake significantly. With 30 s of insonification, the proportion of SPIO positive cells significantly increased with the PNP (i.e., from ~10% at 40 kPa to ~16% at 160 kPa). At the same time, cell viability (Fig 3B) decreased with both the increasing acoustical pressure and the insonification time. For example at 80 kPa PNP, the cell viability decreased from ~70% for 10 s of insonification to ~60% for 30 s of insonification. For a treatment time of 30 s, the cell viability dropped by nearly two-fold from 40 kPa to 160 kPa PNP. In general, the cell viability remained high for up to 40 kPa PNP. Specifically, insonification for 30 s demonstrated the best SPIO uptake and was selected for further experiments. We did not investigate a longer insonification time because after 30 s all tMB were destroyed (see Fig 2E).

### SPIO incubation time

**Fig 4:**
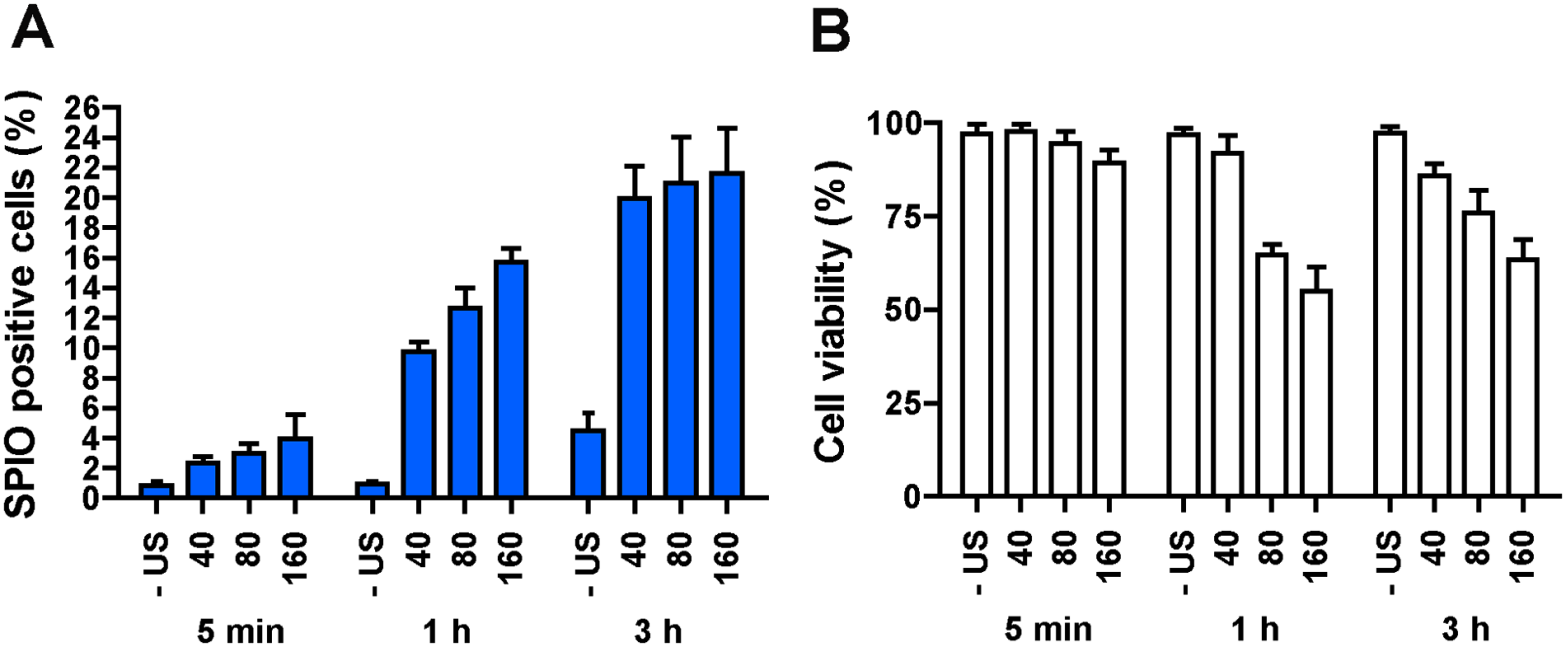
The influence of SPIO incubation time on intracellular SPIO uptake efficiency. (A) SPIO positive cells. (B) Cell viability. HUVECs were treated with tMB and no ultrasound (- US) or ultrasound at varying PNP (40, 80, or 160 kPa) for 30 s. SPIO were added 5 min before insonification (-5 min). Incubation time after SPIO addition was varied from 5 min to 1 h and 3 h.

The influence of the SPIO incubation time (5 min, 1 h or 3 h) after the treatment with ultrasound and tMB on SPIO uptake and cell viability is illustrated in Fig 4. In general, SPIO uptake increased prominently with the incubation time, for example for 180 kPa PNP SPIO uptake increased from below ~4% to ~22%. The largest ratio between control and treated uptake was at 1 h of incubation for all PNPs. Cell viability (Fig 4B) remained high (>75%) at 40 kPa PNP for all incubation times. It decreased with the pressure (80–160 kPa PNP) for both 1 h and 3 h of incubation time. A longer incubation time did not lower cell viability, as cell viability was slightly higher after 3 h of incubation than after 1 h of incubation. Based on the results from this experiment, 1 h of incubation with SPIO was selected for further investigations.

### SPIO addition time

**Fig 5:**
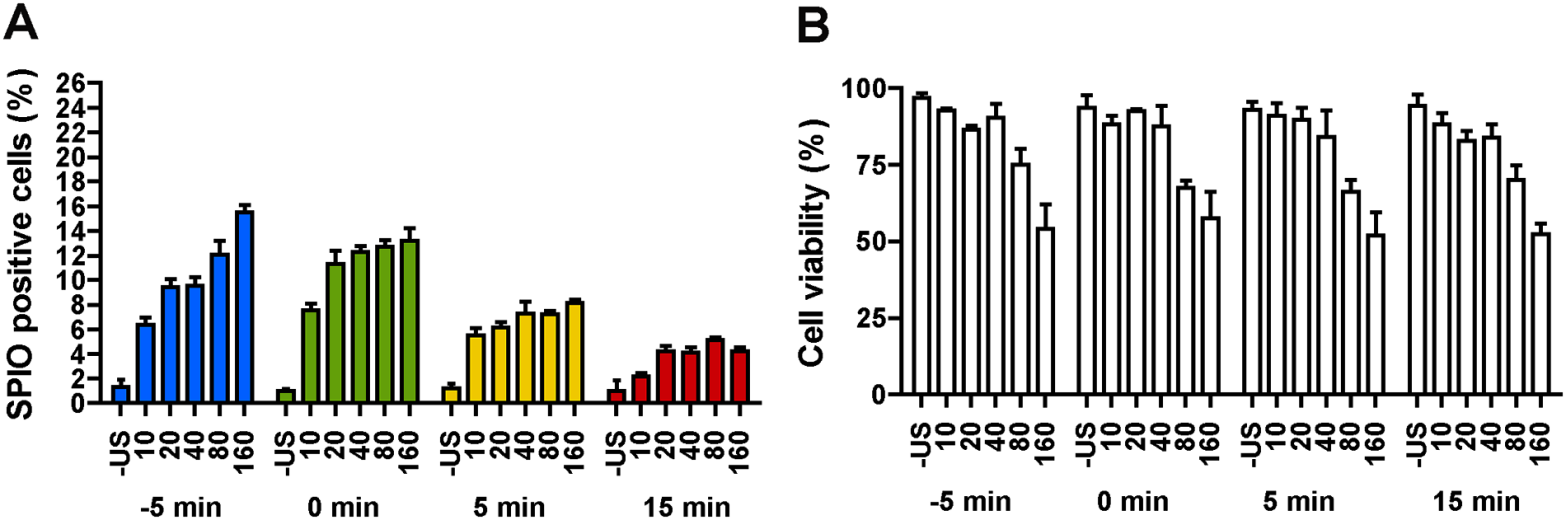
The influence of SPIO addition time on intracellular SPIO uptake efficiency. (A) SPIO positive cells. (B) Cell viability. HUVECs were treated with tMB and no ultrasound (- US) or ultrasound at varying PNP (10, 20, 40, 80, or 160 kPa) for 30 s; SPIO were added 5 min before insonification (-5 min), just before insonification (0 min), 5 min after or 15 min after insonification; 1 h of incubation after SPIO addition.

In Fig 5 the percentage of SPIO positive cells and cell viability are plotted for four different times between insonification and SPIO addition for different acoustic PNP. Similar to the previous observations, no ultrasound application resulted in less than 2% of SPIO positive cells. In addition, ultrasound application without tMBs present showed no significant difference in SPIO uptake in comparison to the control without ultrasound application for all studied PNP when the SPIO were added 5 min prior to insonification. For both additions of SPIO at 5 min prior (-5 min) and just before (0 min) the ultrasound application, we obtained a relatively large percentage of SPIO positive cells (~>10%) for acoustic pressures above 20 kPa PNP. Moreover, the percentage of SPIO positive cells increased up to ~12–15% with higher PNP for SPIO addition before the insonification. In contrast, SPIO addition at 5 and 15 min after ultrasound application resulted in much lower SPIO uptake (<8%). Similarly, the cell viability remained above 50% for all settings.

### SPIO cell labeling

**Fig 6:**
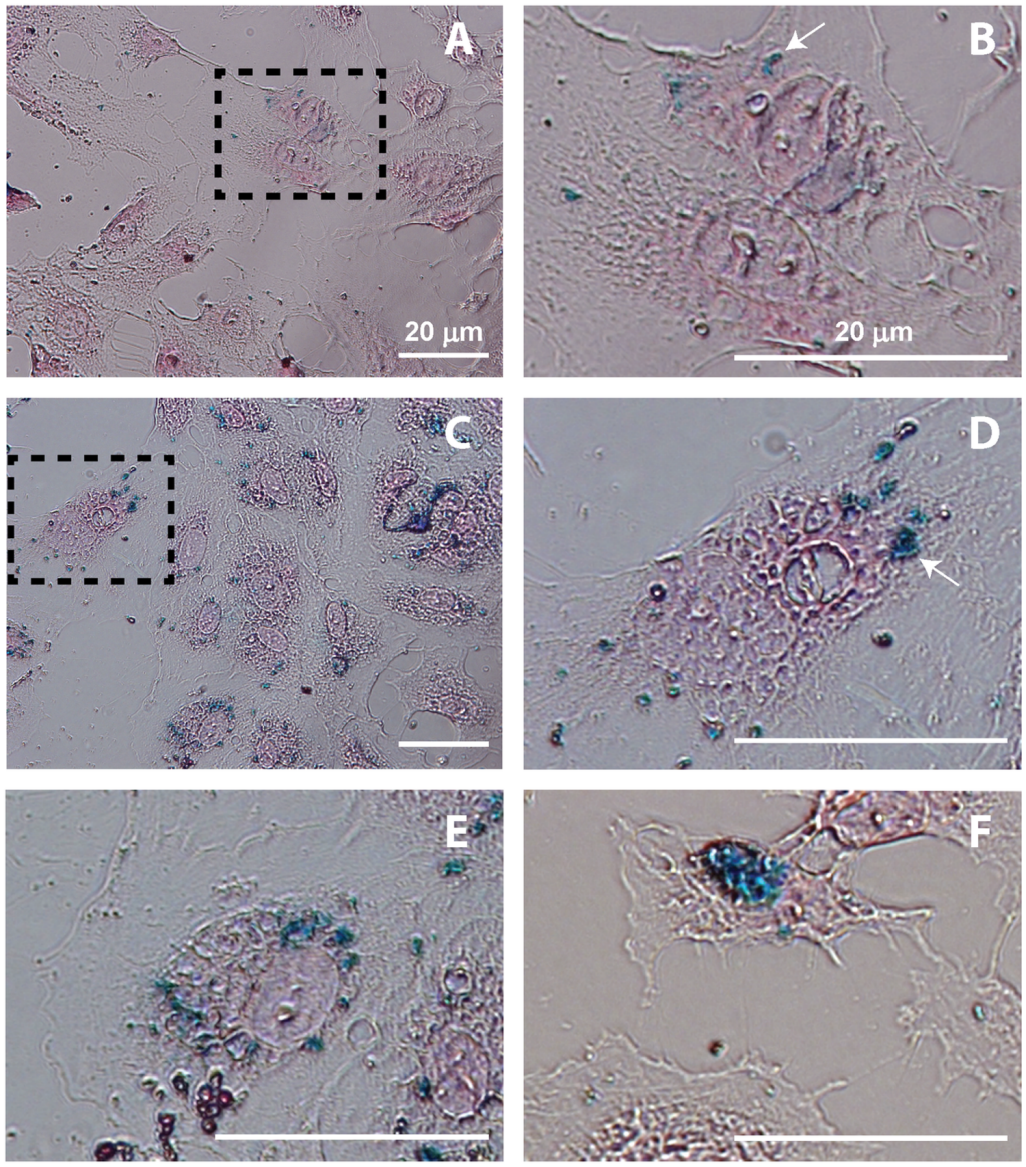
SPIO cell labeling. Prussian Blue staining of SPIO uptake in (A, B) the control (i.e. no ultrasound, 1 h after incubation) and in (C—F) the ultrasound and tMB treated HUVECs at different treatment conditions. C,D,E: 80 kPa PNP, SPIO added at -5 min, 30 s insonification, 1 h incubation after SPIO addition; F: 160 kPa PNP, SPIO added at 15 min, 30 s insonification, 1 h incubation after SPIO addition. B and D are zoomed in from A and C as illustrated by the dashed rectangles. In B and D, one example of a SPIO aggregate is indicated by an arrow.

In the absence of ultrasound, intracellularly incorporated SPIO were detected as small iron particle aggregates distributed in the cytoplasm (Fig 6A and 6B). Ultrasound and tMB treated cells demonstrated much higher SPIO uptake as shown in Fig 6C–6G. Iron particles were detected as aggregates of different sizes in the cytoplasm (Fig 6C and 6D). Other typical individual examples of intracellular SPIO distribution patterns after ultrasound and tMB treatment are shown in Fig 6E and 6F. These staining patterns included distribution of aggregates varying in size and blue intensity throughout the cytoplasm (Fig 6E) and one large aggregate mainly located near the nucleus (Fig 6F) having a higher blue intensity in comparison to the aggregates in Fig 6E. The intensity differences of the blue stain suggest different concentrations of SPIO particles.

## Discussion

Tracking of endothelial cells is important for cancer and cardiovascular disease. There are several ways of SPIO cell labeling *in vitro* [22, 54]. Most of these techniques require different transfection agents, which cannot be used *in vivo* due to the associated high toxicity and systemic effects. We therefore studied a technique, based on ultrasound-activated ultrasound contrast agents that will be compatible for *in vivo* use. The SPIO uptake was dependent on multiple factors, including the ultrasound settings, the time of SPIO addition, and the incubation time of SPIO with cells after the ultrasound treatment. Optimal labeling at 1 MHz ultrasound frequency was observed when the ultrasound parameters were 40 kPa peak negative pressure (MI 0.04), 10,000 cycles and repetition rate of 20 Hz, applied for 30 s when SPIO were added at 0 min. Compared to the control, this resulted in an approximate 12 times increase of SPIO uptake with 85% cell viability.

### Microbubble dynamics

We found an increasing trend of both SPIO positive cells and cell death with the acoustic PNP increase. Note that we studied acoustic pressures up to 160 kPa PNP, a regime in which the amplitude of radial lipid-coated microbubble oscillations increases with pressure [55, 56]. A previous study by Vos et al. [57] has reported that highly non-spherical microbubble vibrations can be induced at pressures as low as 140 kPa PNP for lipid-coated microbubbles at resonance. In this regime, the acoustic streaming generated by oscillating microbubbles and the produced shear stresses [58–61] can be one of the mechanisms for enhanced permeability of the cell membrane [26]. As was expected, the total duration of insonification also showed an effect on the SPIO uptake (Fig 3A). It has been reported that at 1 MHz frequency at a low mechanical index (MI < 0.1), a lipid-coated microbubble can repeatedly oscillate for thousands of cycles; while at higher MI microbubbles are destroyed within about 100 μs (i.e. 100 cycles) irrespective of pulse length [62]. We indeed observed microbubbles still present up to 30 s at 80 kPa PNP (MI = 0.08) (Fig 2). The improved uptake with prolonged insonification may be related with the persistent effect produced by microstreaming generated by microbubble vibrations as formulated earlier. Moreover, we noticed displacement of tMB with subsequent microbubble clustering and merging driven by secondary radiation force over the prolonged burst, as illustrated by Fig 2. These findings are in line with our previous study where tMB bound to endothelial cells also displaced, clustered, and merged by insonification at 1 MHz, albeit for a single burst of up to 50,000 cycles [63]. Detachment of bound lipid-coated tMB has been reported to be due to the attractive secondary Bjerknes force between two tMB [64, 65]. Aggregation of detached tMB forming bigger microbubble clusters may have influenced their oscillation dynamics as larger microbubbles have a lower resonance frequency than individual small microbubbles [66]. At a low driving frequency (for example 1 MHz as applied in this study), microbubble clusters are expected to have a higher amplitude of oscillation as they will be closer to resonance, which could have contributed to the enhanced SPIO uptake.

### SPIO uptake

In our study, HUVECs showed ~1% natural uptake after 1 h of incubation, and this value increased to ~5% after 3 h (Fig 4A). Although the natural uptake of SPIO by HUVECs was previously reported by van Tiel et al. [67], this percentage of labeled cells is not sufficient for cell tracking. Treatment of HUVECs with ultrasound and tMB led to a dramatic increase of ~10-fold in SPIO uptake after 1 h incubation (Fig 4A). On the other hand, cell viability decreased between 5 min and 1 h incubation time (Fig 4B), suggesting that instantaneous cell death (i.e. irreversible sonoporation due to large pores) is less prominent than induced cell death. Induced cell death could occur via the apoptotic pathway, a process that takes time [68, 69], that can be activated by ultrasound and microbubbles as previously reported by others [70–72]. We also observed that the SPIO labeling efficiency was influenced by the SPIO addition time in respect to the time of treatment with tMB and ultrasound (Fig 5). We observed the highest efficacy when SPIO were added with the tMB (0 min) for acoustic PNPs up to 80 kPa. When SPIO were added 5 or 15 min after treatment, SPIO uptake was lower, but still significantly higher (more than fivefold at 5 min) than natural uptake. This may suggest stimulated endocytosis as uptake mechanism rather than sonoporation, since resealing of pores created by ultrasound activated microbubbles has been reported on a relatively short time scale of up to a minute [28, 73]. On the other hand, our results may also suggest that both uptake by stimulated endocytosis and pore formation occurred when SPIO were added 5 min before or just before (0 min) treatment (Fig 5A). This is supported by four earlier ultrasound contrast agent studies [46, 47, 74, 75] as they reported uptake by both pore formation and endocytosis using similar (1 MHz [46, 47, 74, 75]) and different (0.3 MHz [74]) acoustic settings. Meijering et al. [46] and De Cock et al. [47] also addressed the influence of particle size on the uptake route: for dextrans larger than ~17 nm in radius, the primary uptake route was endocytosis instead of pore formation. On the other hand, pores of 1 nm in size [76] to > 100 μm^2^ in area [28] have been observed. However, induced pores >100 μm^2^ do not tend to reseal [28] which likely leads to cell death. This could explain why higher acoustic pressures (i.e., ≥ 80 kPa) in our study corresponded to increased cell death. Pores < 100 μm^2^ can still reseal, according to a recent study [28]. The SPIO colloid with low molecular weight dextran coating in Endorem^™^, as used in our study, are 120–180 nm in size [77] so they could enter cells via resealable pores. The reason why Meijering et al. [46] observed dextran particles larger than ~17 nm mainly to be taken up by endocytosis, could be the different type of microbubble used (SonoVue versus tMB in our study) and/or the type of cells studied (bovine aortic endothelial cells versus HUVECs in our study).

The amount of SPIO positive cells was almost two times higher when SPIO were added 5 min instead of 15 min after insonification. This suggests a relatively short temporal window when the therapeutic agent can be actively taken up after microbubble insonification, especially when compared to the study by Yudina et al. who reported a temporal window of 24 h [78]. The difference could be due to the therapeutic compound (small 600 Da molecule Sytox Green versus the 120–180 nm SPIO in our study), the type of microbubble (SonoVue versus tMB in our study), the acoustic settings (ultrasound frequency of 1.5 MHz, 440 kPa PNP, 300 cycles at 1 kHz repetition rate for 30 s versus 1 MHz, ≤160 kPa PNP, 10,000 cycles at 20 Hz for 30 s in our study) and/or the type of cells studied (C6 rat glioma cells versus HUVECs in our study). Recently, the temporal window was reported to be cell type-dependent by the same group [79]. For HUVECs, the temporal was found to be 1 h for the small molecule SYTOX green.

In our study, we defined a cell as SPIO positive when we detected one or more SPIO particles within the cell. It is unlikely that the SPIO particles we detected were extracellular because the cells were washed three times. We observed different labeling patterns, namely differences in the intensity and the intracellular distribution of the SPIO particles, although we did not quantify this degree of uptake. Some cells took up small spots (submicron) of SPIO as dense granules ~2 nm in diameter, while others had large SPIO aggregates in the cytoplasm. This variation in uptake patterns may also suggest uptake by pore formation and endocytosis as SPIO homogeneously distributed in the cytoplasm may suggest pore formation while small aggregates may indicate endocytic uptake. This is supported by different uptake patterns previously reported for dextrans with aggregates verified to be co-localized with endocytic vesicles [46, 47].

As ultrasound contrast agent-mediated SPIO-labeling of endothelial cells is likely faster clinically approved for labeling tumor vasculature, we decided to mimic the tumor vasculature’s compromised endothelial monolayer with poorly connected and sprouting endothelial cells [80] by culturing the HUVECs till 70% confluence. Our findings of effective tMB-mediated SPIO-labeling may therefore not be applicable to a monolayer of HUVECs, i.e. 100% confluence, applicable to vascular grafts. Different ultrasound pressures may be needed for effective tMB-mediated SPIO-labeling HUVECs in a monolayer as cells in a monolayer are more likely in the senescent cell cycle phase (G_0_) and have a more organized cytoskeleton. With respect to sensitivity to ultrasound treatment, cells in the mitotis(M)-phase [81, 82] and synthesis(S)-phase [82] have been reported most sensitive whereas cells in the senescent cell cycle phase were least sensitive. By contrast, another study showed the opposite: cells in the M and S-phases were more resistant to ultrasound treatment [83]. However, these ultrasound sensitivity studies were performed without the presence of MB and on cancer/epithelial cells. These studies may therefore not translate to our study and it needs further investigation whether the amount of cell confluence influences the response to tMB-treatment.

It was previously shown [38] that microbubbles with SPIO incorporated in their coating in combination with ultrasound could lead to an about three fold increase of SPIO labeling of tumor cells *in vitro*, without compromising cell viability. A polymer coated microbubble was used, which has been shown to behave differently than a lipid coated microbubble when exposed to ultrasound. Polymer microbubbles have a stiff coating, which can respond to ultrasound exposure under a high MI (>1) by cracking and releasing the encapsulated gas. On the contrary, lipid coated microbubbles will oscillate at low MI and can also fuse [26]. They can therefore have a more prolonged interaction with cells than polymer microbubbles. This could explain why we found a ~12 fold increase in SPIO labeling compared to the control treatment.

### Clinical implications

From the *in vivo* perspective, using tMB instead of non-tMB is preferable for endothelial cell labeling and drug delivery since tMB can be specifically targeted to diseased endothelial cells [84]. In addition, when ultrasound is applied to bound tMB, the vibrations of the ultrasound-activated tMB will have a direct effect on the cell membrane. This may be the reason why tMB have been shown to be up to ~5 times more effective in stimulating cellular uptake of therapeutics *in vivo* [30]. CD31 used as target in this proof of concept study was chosen as model ligand because it is constitutively expressed on endothelial cell membranes. It can therefore be used to label endothelial cells with SPIO in tissue-engineered valves or vascular grafts *in vitro*. However, CD31 cannot be used *in vivo* as it is expressed throughout the entire vasculature tree [85]. For targeting tMB to tumor vasculature, α_v_β_3_ or vascular endothelial growth factor receptor 2 (VEGFR2) can be used [84]. SPIO uptake by ultrasound-activated tMB *in vivo* is expected as we recently reported that tMB bound to α_v_β_3_ can stimulate endothelial cell drug uptake *in vivo* [50]. VEGFR2 is another biomarker of interest for tMB. BR55, a tMB against VEGFR2, has recently successfully been used in clinical trials for prostate, breast, and ovarian cancer [86, 87]. Our future studies will focus on *in vivo* SPIO labeling as well as *in vivo* MRI tracking of the labeled endothelial cells. For the *in vivo* studies, our *in vitro* acoustic settings will need to be extrapolated taking into account that ultrasound is attenuated by tissue (0.5 dB/cm/MHz [88]) and the microbubble vibration is damped in blood [89]. In our *in vitro* study we incubated the HUVECs for 1 h with the SPIO. As the elimination half-life of Endorem^™^ (Feridex^®^ in the USA) is 2.4 ± 0.2 h in humans and the SPIO are administered as a drip infusion over ~30 min [77, 90], it may be expected that the SPIO will circulate long enough *in vivo* to also achieve the 1 h incubation period. It was shown before that single cell tracking is possible by MRI using iron oxide as the label [13, 14, 16]. The micron-sized paramagnetic iron oxide (MPIO) particles used in one of these studies [13] are ten times bigger (1.6 μm) than SPIO. As it was revealed before [67] that the iron content of cells labeled with SPIO is less (~10 folds) than with MPIO, more cells may have to be labeled by SPIO to be detectable by MRI *in vivo*.

## Conclusion

Our study shows that ultrasound-activated tMB are a promising technique to non-invasively enhance SPIO uptake by endothelial cells. From the current *in vitro* study, we derived optimal ultrasound parameters for SPIO delivery to HUVECs, that is, 40 kPa at 1 MHz (MI 0.04), 10,000 cycles and repetition rate of 20 Hz, applied for 30 s when SPIO were added at 0 min. This setting increased SPIO uptake up to 12 times compared to the control with 85% cell viability.

## Supporting information

S1 Fig Cell viability determined from calcein-AM versus PI staining. Insonification for 30 s for SPIO added at -5, 0, 5, or 15 min in respect to the start of insonification and incubated for 1 h. The acoustic PNP in (A) was 10 kPa, while this was 20 kPa in (B), 40 kPa in (C), 80 kPa in (D), and 160 kPa in (E).
(TIF)

## References

[1] Akbari-Birgani S, Paranjothy T, Zuse A, Janikowski T, Cieslar-Pobuda A, Likus W, et al Cancer stem cells, cancer-initiating cells and methods for their detection. Drug Discov Today. 2016;21(5):836–42. 10.1016/j.drudis.2016.03.004 26976692

[2] Moreira ML, da Costa Medeiros P, de Souza SA, Gutfilen B, Rosado-de-Castro PH. In Vivo Tracking of Cell Therapies for Cardiac Diseases with Nuclear Medicine. Stem Cells Int. 2016;2016:3140120 10.1155/2016/3140120 26880951 PMC4737458

[3] Bernsen MR, Kooiman K, Segbers M, van Leeuwen FW, de Jong M. Biomarkers in preclinical cancer imaging. Eur J Nucl Med Mol Imaging. 2015;42(4):579–96. 10.1007/s00259-014-2980-7 25673052 PMC4348504

[4] Accomasso L, Gallina C, Turinetto V, Giachino C. Stem Cell Tracking with Nanoparticles for Regenerative Medicine Purposes: An Overview. Stem Cells Int. 2016;2016:7920358 10.1155/2016/7920358 26839568 PMC4709786

[5] Liu Z, Li Z. Molecular imaging in tracking tumor-specific cytotoxic T lymphocytes (CTLs). Theranostics. 2014;4(10):990–1001. 10.7150/thno.9268 25157278 PMC4142291

[6] Hong H, Yang Y, Zhang Y, Cai W. Non-invasive cell tracking in cancer and cancer therapy. Curr Top Med Chem. 2010;10(12):1237–48. 20388105 10.2174/156802610791384234 PMC2916057

[7] Gimi B, Mori N, Ackerstaff E, Frost EE, Bulte JW, Bhujwalla ZM. Noninvasive MRI of endothelial cell response to human breast cancer cells. Neoplasia. 2006;8(3):207–13. 10.1593/neo.05547 16611414 PMC1578524

[8] Xiong L, Cao F, Cao X, Guo Y, Zhang Y, Cai X. Long-term-stable near-infrared polymer dots with ultrasmall size and narrow-band emission for imaging tumor vasculature in vivo. Bioconjug Chem. 2015;26(5):817–21. 10.1021/acs.bioconjchem.5b00163 25928072

[9] Ramaswamy S, Schornack PA, Smelko AG, Boronyak SM, Ivanova J, Mayer JE Jr, et al Superparamagnetic iron oxide (SPIO) labeling efficiency and subsequent MRI tracking of native cell populations pertinent to pulmonary heart valve tissue engineering studies. NMR Biomed. 2012;25(3):410–7. 10.1002/nbm.1642 22351640

[10] Fayol D, Le Visage C, Ino J, Gazeau F, Letourneur D, Wilhelm C. Design of biomimetic vascular grafts with magnetic endothelial patterning. Cell Transplant. 2013;22(11):2105–18. 10.3727/096368912X661300 23295155

[11] Fu Y, Azene N, Xu Y, Kraitchman DL. Tracking stem cells for cardiovascular applications in vivo: focus on imaging techniques. Imaging Med. 2011;3(4):473–86 22287982 10.2217/iim.11.33 PMC3265127

[12] Youn H, Hong KJ. In vivo non invasive molecular imaging for immune cell tracking in small animals. Immune Netw. 2012;12(6):223–9. 10.4110/in.2012.12.6.223 23396713 PMC3566416

[13] Shapiro EM, Sharer K, Skrtic S, Koretsky AP. In vivo detection of single cells by MRI. Magn Reson Med. 2006;55(2):242–9. 10.1002/mrm.20718 16416426

[14] Lee N, Kim H, Choi SH, Park M, Kim D, Kim HC, et al Magnetosome-like ferrimagnetic iron oxide nanocubes for highly sensitive MRI of single cells and transplanted pancreatic islets. Proc Natl Acad Sci USA. 2011;108(7):2662–7. 10.1073/pnas.1016409108 21282616 PMC3041081

[15] Ngen EJ, Artemov D. Advances in Monitoring Cell-Based Therapies with Magnetic Resonance Imaging: Future Perspectives. Int J Mol Sci. 2017;18(1). 10.3390/ijms18010198 PMC5297829 28106829

[16] Zhang Z, van den Bos EJ, Wielopolski PA, de Jong-Popijus M, Bernsen MR, Duncker DJ, et al In vitro imaging of single living human umbilical vein endothelial cells with a clinical 3.0-T MRI scanner. MAGMA. 2005;18(4):175–85. 10.1007/s10334-005-0108-6 16096808

[17] Gamarra LF, Brito GES, Pontuschka WM, Amaro E, Parma AHC, Goya GF. Biocompatible superparamagnetic iron oxide nanoparticles used for contrast agents: a structural and magnetic study. J Magn Magn Mater. 2005;289(0):439–41.

[18] Neuwelt A, Sidhu N, Hu CA, Mlady G, Eberhardt SC, Sillerud LO. Iron-based superparamagnetic nanoparticle contrast agents for MRI of infection and inflammation. AJR Am J Roentgenol. 2015;204(3):W302–13. 10.2214/AJR.14.12733 25714316 PMC4395032

[19] Bernsen MR, Guenoun J, Van Tiel ST, Krestin GP. Nanoparticles and clinically applicable cell tracking. Brit J Radiol. 2015;88(1054). 10.1259/bjr.20150375 PMC4730978 26248872

[20] Reddy LH. Drug delivery to tumours: recent strategies. J Pharm Pharmacol. 2005;57(10):1231–42. 10.1211/jpp.57.10.0001 16259751

[21] Reddy LH, Arias JL, Nicolas J, Couvreur P. Magnetic nanoparticles: design and characterization, toxicity and biocompatibility, pharmaceutical and biomedical applications. Chem Rev. 2012;112(11):5818–78. 10.1021/cr300068p 23043508

[22] Frank JA, Anderson SA, Kalsih H, Jordan EK, Lewis BK, Yocum GT, et al Methods for magnetically labeling stem and other cells for detection by in vivo magnetic resonance imaging. Cytotherapy. 2004;6(6):621–5. 15773025 10.1080/14653240410005267-1

[23] Rodriguez-Porcel M, Kronenberg MW, Henry TD, Traverse JH, Pepine CJ, Ellis SG, et al Cell tracking and the development of cell-based therapies: a view from the Cardiovascular Cell Therapy Research Network. JACC Cardiovasc Imaging. 2012;5(5):559–65. 10.1016/j.jcmg.2011.12.018 22595165 PMC3632261

[24] Ferrara K, Pollard R, Borden M. Ultrasound microbubble contrast agents: fundamentals and application to gene and drug delivery. Annu Rev Biomed Eng. 2007;9:415–47. 10.1146/annurev.bioeng.8.061505.095852 17651012

[25] Lentacker I, De Cock I, Deckers R, De Smedt S, Moonen CTW. Understanding ultrasound induced sonporation: Definitions and underlying mechanisms. Adv Drug Deliver Rev. 2014. 10.1016/j.addr.2013.11.008 24270006

[26] Kooiman K, Vos HJ, Versluis M, de Jong N. Acoustic behavior of microbubbles and implications for drug delivery. Adv Drug Deliver Rev. 2014;72C:28–48. 10.1016/j.addr.2014.03.003 24667643

[27] Sutton JT, Haworth KJ, Pyne-Geithman G, Holland CK. Ultrasound-mediated drug delivery for cardiovascular disease. Expert Opin Drug Deliv. 2013;10(5):573–92. 10.1517/17425247.2013.772578 23448121 PMC4026001

[28] Hu Y, Wan JM, Yu AC. Membrane perforation and recovery dynamics in microbubble-mediated sonoporation. Ultrasound Med Biol. 2013;39(12):2393–405. 10.1016/j.ultrasmedbio.2013.08.003 24063956

[29] Yan F, Li X, Jin Q, Jiang C, Zhang Z, Ling T, et al Therapeutic ultrasonic microbubbles carrying paclitaxel and LyP-1 peptide: preparation, characterization and application to ultrasound-assisted chemotherapy in breast cancer cells. Ultrasound Med Biol. 2011;37(5):768–79. 10.1016/j.ultrasmedbio.2011.02.006 21458148

[30] Xie A, Belcik T, Qi Y, Morgan TK, Champaneri SA, Taylor S, et al Ultrasound-Mediated Vascular Gene Transfection by Cavitation of Endothelial-Targeted Cationic Microbubbles. JACC Cardiovasc Imag. 2012;5(12):1253–62. 10.1016/j.jcmg.2012.05.017 PMC3682923 23236976

[31] Klibanov AL. Preparation of targeted microbubbles: ultrasound contrast agents for molecular imaging. Med Biol Eng Comput. 2009;47(8):875–82. 10.1007/s11517-009-0498-0 19517153

[32] van Rooij T, Daeichin V, Skachkov I, de Jong N, Kooiman K. Targeted ultrasound contrast agents for ultrasound molecular imaging and therapy. Int J Hyperthermia. 2015;31(2):90–106. 10.3109/02656736.2014.997809 25707815

[33] Liu HL, Chen PY, Yang HW, Wu JS, Tseng IC, Ma YJ, et al In vivo MR quantification of superparamagnetic iron oxide nanoparticle leakage during low-frequency-ultrasound-induced blood-brain barrier opening in swine. J Magn Reson Imaging. 2011;34(6):1313–24. 10.1002/jmri.22697 21965168

[34] Fan CH, Ting CY, Lin HJ, Wang CH, Liu HL, Yen TC, et al SPIO-conjugated, doxorubicin-loaded microbubbles for concurrent MRI and focused-ultrasound enhanced brain-tumor drug delivery. Biomaterials. 2013;34(14):3706–15. 10.1016/j.biomaterials.2013.01.099 23433776

[35] Fan CH, Cheng YH, Ting CY, Ho YJ, Hsu PH, Liu HL, et al Ultrasound/Magnetic Targeting with SPIO-DOX-Microbubble Complex for Image-Guided Drug Delivery in Brain Tumors. Theranostics. 2016;6(10):1542–56. 10.7150/thno.15297 27446489 PMC4955054

[36] Gao WQ, Li X, Liu PC, Bai ZY, Zhong YF, Han HB, et al Investigation in vivo of effect of ultrasound-mediated microbubble destruction on entrance of feridex into the aortal wall. Zhonghua yi xue za zhi. 2009;89(39):2797–801. 20137608

[37] Liu ZY, Wang Y, Liang CH, Li XH, Wang GY, Liu HJ, et al In vitro labeling of mesenchymal stem cells with superparamagnetic iron oxide by means of microbubble-enhanced US exposure: initial experience. Radiology. 2009;253(1):153–9. 10.1148/radiol.2531081974 19710004

[38] Yang F, Zhang M, He W, Chen P, Cai X, Yang L, et al Controlled release of Fe3O4 nanoparticles in encapsulated microbubbles to tumor cells via sonoporation and associated cellular bioeffects. Small. 2011;7(7):902–10. 10.1002/smll.201002185 21374806

[39] Correas JM, Helenon O, Pourcelot L, Moreau JF. Ultrasound contrast agents. Examples of blood pool agents. Acta Radiol Suppl. 1997;412:101–12. 9240088

[40] Greis C. Ultrasound contrast agents as markers of vascularity and microcirculation. Clin Hemorheol Microcirc. 2009;43(1):1–9. 10.3233/CH-2009-1216 19713597

[41] Eguchi S, Takatsuki M, Hidaka M, Soyama A, Tomonaga T, Muraoka I, et al Predictor for histological microvascular invasion of hepatocellular carcinoma: a lesson from 229 consecutive cases of curative liver resection. World J Surg. 2010;34(5):1034–8. 10.1007/s00268-010-0424-5 20127241

[42] Sternberg A, Amar M, Alfici R, Groisman G. Conclusions from a study of venous invasion in stage IV colorectal adenocarcinoma. J Clin Pathol. 2002;55(1):17–21. 11825918 10.1136/jcp.55.1.17 PMC1769571

[43] Pu C, Chang S, Sun J, Zhu S, Liu H, Zhu Y, et al Ultrasound-mediated destruction of LHRHa-targeted and paclitaxel-loaded lipid microbubbles for the treatment of intraperitoneal ovarian cancer xenografts. Mol Pharm. 2014;11(1):49–58. 10.1021/mp400523h 24237050 PMC3899929

[44] Newman PJ, Newman DK. Signal transduction pathways mediated by PECAM-1: new roles for an old molecule in platelet and vascular cell biology. Arterioscl Thromb Vas. 2003;23(6):953–64. 10.1161/01.ATV.0000071347.69358.D9 12689916

[45] Jin LF, Li F, Wang HP, Wei F, Qin P, Du LF. Ultrasound targeted microbubble destruction stimulates cellular endocytosis in facilitation of adeno-associated virus delivery. Int J Mol Sci. 2013;14(5):9737–50. 10.3390/ijms14059737 23652832 PMC3676809

[46] Meijering BD, Juffermans LJ, van Wamel A, Henning RH, Zuhorn IS, Emmer M, et al Ultrasound and microbubble-targeted delivery of macromolecules is regulated by induction of endocytosis and pore formation. Circ Res. 2009;104(5):679–87. 10.1161/CIRCRESAHA.108.183806 19168443

[47] De Cock I, Zagato E, Braeckmans K, Luan Y, de Jong N, De Smedt SC, et al Ultrasound and microbubble mediated drug delivery: acoustic pressure as determinant for uptake via membrane pores or endocytosis. J Control Release. 2015;197:20–8. 10.1016/j.jconrel.2014.10.031 25449801

[48] Kooiman K, Foppen-Harteveld M, van der Steen AFW, de Jong N. Sonoporation of endothelial cells by vibrating targeted microbubbles. J Control Release. 2011;154(1):35–41. 10.1016/j.jconrel.2011.04.008 21514333

[49] Klibanov AL, Rasche PT, Hughes MS, Wojdyla JK, Galen KP, Wible JH Jr, et al Detection of individual microbubbles of ultrasound contrast agents: imaging of free-floating and targeted bubbles. Invest Radiol. 2004;39(3):187–95. 15076011 10.1097/01.rli.0000115926.96796.75

[50] Skachkov I, Luan Y, van der Steen AF, de Jong N, Kooiman K. Targeted microbubble mediated sonoporation of endothelial cells in vivo. IEEE Trans Ultrason Ferroelectr Freq Control. 2014;61(10):1661–7. 10.1109/TUFFC.2014.006440 25265175

[51] Lindner JR, Song J, Christiansen J, Klibanov AL, Xu F, Ley K. Ultrasound assessment of inflammation and renal tissue injury with microbubbles targeted to P-selectin. Circulation. 2001;104(17):2107–12. 11673354 10.1161/hc4201.097061

[52] Clark G. Staining procedures used by the Biological Stain Commission. 3 ed: Published for the Biological Stain Commission by Williams & Wilkins; 1973.

[53] Rasband WS. ImageJ. Bethesda, Maryland, USA: U.S. National Institutes of Health; 1997–2018.

[54] Bernsen MR, Moelker AD, Wielopolski PA, van Tiel ST, Krestin GP. Labelling of mammalian cells for visualisation by MRI. Eur Radiol. 2010;20(2):255–74. 10.1007/s00330-009-1540-1 19672602

[55] Vos HJ, Dollet B, Bosch JG, Versluis M, de Jong N. Nonspherical vibrations of microbubbles in contact with a wall: a pilot study at low mechanical index. Ultrasound Med Biol. 2008;34(4):685–8. 10.1016/j.ultrasmedbio.2007.10.001 18077080

[56] Overvelde M, Garbin V, Dollet B, de Jong N, Lohse D, Versluis M. Dynamics of coated microbubbles adherent to a wall. Ultrasound Med Biol. 2011;37(9):1500–8. 10.1016/j.ultrasmedbio.2011.05.025 21816289

[57] Vos HJ, Dollet B, Versluis M, de Jong N. Nonspherical Shape Oscillations of Coated Microbubbles in Contact with a Wall. Ultrasound Med Biol. 2011;37(6):935–48. 10.1016/j.ultrasmedbio.2011.02.013 21601137

[58] Prentice P, Cuschierp A, Dholakia K, Prausnitz M, Campbell P. Membrane disruption by optically controlled microbubble cavitation. Nat Phys. 2005;1(2):107–10.

[59] Mukherjee D, Wong J, Griffin B, Ellis SG, Porter T, Sen S, et al Ten-fold augmentation of endothelial uptake of vascular endothelial growth factor with ultrasound after systemic administration. J Am Coll Cardiol. 2000;35(6):1678–86. 10807476 10.1016/s0735-1097(00)00575-1

[60] Ohl CD, Arora M, Ikink R, de Jong N, Versluis M, Delius M, et al Sonoporation from jetting cavitation bubbles. Biophys J. 2006;91(11):4285–95. 10.1529/biophysj.105.075366 16950843 PMC1635670

[61] Ooi A, Tho P, Manasseh R. Cavitation microstreaming patterns in single and multiple bubble systems. J Acoust Soc Am. 2007;122(5):3051.

[62] Mannaris C, Averkiou MA. Investigation of microbubble response to long pulses used in ultrasound-enhanced drug delivery. Ultrasound Med Biol. 2012;38(4):681–91. 10.1016/j.ultrasmedbio.2011.12.018 22341047

[63] van Rooij T, Skachkov I, Beekers I, Lattwein KR, Voorneveld JD, Kokhuis TJ, et al Viability of endothelial cells after ultrasound-mediated sonoporation: Influence of targeting, oscillation, and displacement of microbubbles. J Control Release. 2016;238:197–211. 10.1016/j.jconrel.2016.07.037 27469471

[64] Kokhuis TJ, Garbin V, Kooiman K, Naaijkens BA, Juffermans LJ, Kamp O, et al Secondary Bjerknes forces deform targeted microbubbles. Ultrasound Med Biol. 2013;39(3):490–506. 10.1016/j.ultrasmedbio.2012.09.025 23347643

[65] Garbin V, Dollet B, Overvelde M, Cojoc D, Di Fabrizio E, van Wijngaarden L, et al History force on coated microbubbles propelled by ultrasound. Phys Fluids. 2009;21(9):092003.

[66] Dayton PA, Morgan KE, Klibanov AL, Brandenburger GH, Ferrara KW. Optical and acoustical observations of the effects of ultrasound on contrast agents. IEEE Trans Ultrason Ferroelectr Freq Control. 1999;46(1):220–32. 10.1109/58.741536 18238417

[67] van Tiel ST, Wielopolski PA, Houston GC, Krestin GP, Bernsen MR. Variations in labeling protocol influence incorporation, distribution and retention of iron oxide nanoparticles into human umbilical vein endothelial cells. Contrast Media Mol Imaging. 2010;5(5):247–57. 10.1002/cmmi.379 20973110

[68] Green DR. Apoptotic pathways: ten minutes to dead. Cell. 2005;121(5):671–4. 10.1016/j.cell.2005.05.019 15935754

[69] Elmore S. Apoptosis: a review of programmed cell death. Toxicol Pathol. 2007;35(4):495–516. 10.1080/01926230701320337 17562483 PMC2117903

[70] Feril LB Jr, Kondo T, Zhao QL, Ogawa R, Tachibana K, Kudo N, et al Enhancement of ultrasound-induced apoptosis and cell lysis by echo-contrast agents. Ultrasound Med Biol. 2003;29(2):331–7. 12659921 10.1016/s0301-5629(02)00700-7

[71] Zhong W, Sit WH, Wan JM, Yu AC. Sonoporation induces apoptosis and cell cycle arrest in human promyelocytic leukemia cells. Ultrasound Med Biol. 2011;37(12):2149–59. 10.1016/j.ultrasmedbio.2011.09.012 22033133

[72] Miller DL, Dou C. Induction of apoptosis in sonoporation and ultrasonic gene transfer. Ultrasound Med Biol. 2009;35(1):144–54. 10.1016/j.ultrasmedbio.2008.06.007 18723272 PMC2642595

[73] Fan Z, Liu H, Mayer M, Deng CX. Spatiotemporally controlled single cell sonoporation. Proc Natl Acad Sci USA. 2012;109(41):16486–91. 10.1073/pnas.1208198109 23012425 PMC3478613

[74] Afadzi M, Strand SP, Nilssen EA, Masoy SE, Johansen TF, Hansen R, et al Mechanisms of the ultrasound-mediated intracellular delivery of liposomes and dextrans. IEEE Trans Ultrason Ferroelectr Freq Control. 2013;60(1):21–33. 10.1109/TUFFC.2013.2534 23287910

[75] Zeghimi A, Escoffre JM, Bouakaz A. Role of endocytosis in sonoporation-mediated membrane permeabilization and uptake of small molecules: a electron microscopy study. Phys Biol. 2015;12(6):066007 10.1088/1478-3975/12/6/066007 26599283

[76] Yang F, Gu N, Chen D, Xi X, Zhang D, Li Y, et al Experimental study on cell self-sealing during sonoporation. J Control Release. 2008;131(3):205–10. 10.1016/j.jconrel.2008.07.038 18727944

[77] Wang YX. Superparamagnetic iron oxide based MRI contrast agents: Current status of clinical application. Quant Imaging Med Surg. 2011;1(1):35–40. 10.3978/j.issn.2223-4292.2011.08.03 23256052 PMC3496483

[78] Yudina A, Lepetit-Coiffe M, Moonen CT. Evaluation of the temporal window for drug delivery following ultrasound-mediated membrane permeability enhancement. Mol Imaging Biol. 2011;13(2):239–49. 10.1007/s11307-010-0346-5 20521134

[79] Lammertink B, Deckers R, Storm G, Moonen C, Bos C. Duration of ultrasound-mediated enhanced plasma membrane permeability. Int J Pharm. 2015;482(1–2):92–8. 10.1016/j.ijpharm.2014.12.013 25497443

[80] Hashizume H, Baluk P, Morikawa S, McLean JW, Thurston G, Roberge S, et al Openings between defective endothelial cells explain tumor vessel leakiness. Am J Pathol. 2000;156(4):1363–80. 10.1016/S0002-9440(10)65006-7 10751361 PMC1876882

[81] Clarke PR, Hill CR. Biological action of ultrasound in relation to the cell cycle. Exp Cell Res. 1969;58(2):443–4. 5408620 10.1016/0014-4827(69)90529-1

[82] Hrazdira I, Skorpikova J, Dolnikova M. Ultrasonically induced alterations of cultured tumour cells. Eur J Ultrasound. 1998;8(1):43–9. 9795012 10.1016/s0929-8266(98)00049-4

[83] Fu YK, Miller MW, Lange CS, Griffiths TD, Kaufman GE. Ultrasound lethality to synchronous and asynchronous Chinese hamster V-79 cells. Ultrasound Med Biol. 1980;6(1):39–46. 6989076 10.1016/0301-5629(80)90062-9

[84] Abou-Elkacem L, Bachawal SV, Willmann JK. Ultrasound molecular imaging: Moving toward clinical translation. Eur J Radiol. 2015;84(9):1685–93. 10.1016/j.ejrad.2015.03.016 25851932 PMC4545409

[85] DeLisser HM, Newman PJ, Albelda SM. Molecular and functional aspects of PECAM-1/CD31. Immunol Today. 1994;15(10):490–5. 10.1016/0167-5699(94)90195-3 7945775

[86] Smeenge M, Tranquart F, Mannaerts CK, de Reijke TM, van de Vijver MJ, Laguna MP, et al First-in-Human Ultrasound Molecular Imaging With a VEGFR2-Specific Ultrasound Molecular Contrast Agent (BR55) in Prostate Cancer: A Safety and Feasibility Pilot Study. Invest Radiol. 2017;52(7):419–27. 10.1097/RLI.0000000000000362 28257340

[87] Willmann JK, Bonomo L, Carla Testa A, Rinaldi P, Rindi G, Valluru KS, et al Ultrasound Molecular Imaging With BR55 in Patients With Breast and Ovarian Lesions: First-in-Human Results. J Clin Oncol. 2017;35(19):2133–40. 10.1200/JCO.2016.70.8594 28291391 PMC5493049

[88] Mast TD. Empirical relationships between acoustic parameters in human soft tissue. Acoust Res Lett Online. 2000;1:37–42.

[89] de Jong N, Bouakaz A, Frinking P. Basic acoustic properties of microbubbles. Echocardiography. 2002;19(3):229–40. 12022933 10.1046/j.1540-8175.2002.00229.x

[90] FDA. Feridex I.V.^®^ label [http://www.fda.gov/ohrms/dockets/ac/05/briefing/2005-4095B1_02_14-FDA-Tab-7-9.pdf]. [http://www.fda.gov/ohrms/dockets/ac/05/briefing/2005-4095B1_02_14-FDA-Tab-7-9.pdf:[http://www.fda.gov/ohrms/dockets/ac/05/briefing/2005-4095B1_02_14-FDA-Tab-7-9.pdf.

