# Supplementary figures and images for: SPIO labeling of endothelial cells using ultrasound and targeted microbubbles at diagnostic pressures
Source: PLoS One. 2018 Sep 20;13(9):e0204354. doi: 10.1371/journal.pone.0204354 (PMC6147550; doi:10.1371/journal.pone.0204354)

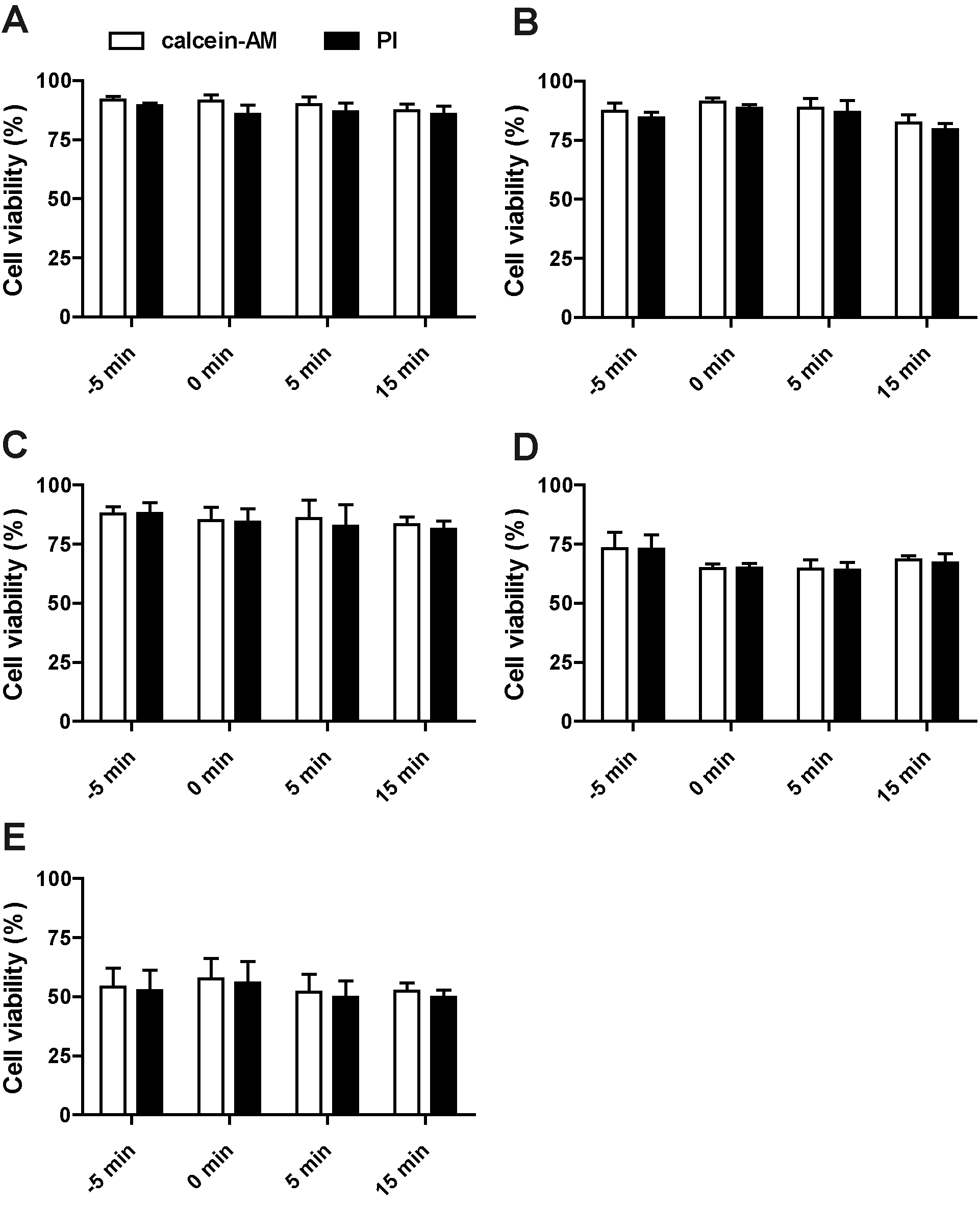

Supplement: S1 Fig — Insonification for 30 s for SPIO added at -5, 0, 5, or 15 min in respect to the start of insonification and incubated for 1 h. The acoustic PNP in (A) was 10 kPa, while this was 20 kPa in (B), 40 kPa in (C), 80 kPa in (D), and 160 kPa in (E). (TIF) [file pone.0204354.s001.tif]
